# Breaking the crosstalk of the cellular tumorigenic network: Hypothesis for addressing resistances to targeted therapies in advanced NSCLC

**DOI:** 10.18632/oncotarget.16674

**Published:** 2017-03-29

**Authors:** Stefan Langhammer, Joachim Scheerer

**Affiliations:** ^1^ Life Science Consulting, Hirschweg, Burgwedel, Germany

**Keywords:** NSCLC, targeted therapy, EGFR, angiogenesis

## Abstract

In the light of current treatment developments for non-small cell lung cancer (NSCLC), the idea of a plastic cellular tumorigenic network bound by key paracrine signaling pathways mediating resistances to targeted therapies is brought forward. Based on a review of available preclinical and clinical data in NSCLC combinational approaches to address drivers of this network with marketed drugs are discussed. Five criteria for selecting drug combination regimens aiming at its disruption and thereby overcoming resistances are postulated.

## THE HOLISTIC CONCEPT OF TUMOR BIOLOGY

In 2011 Hanahan and Weinberg updated their concept “Hallmarks of Cancer” by adding two further aspects of tumor biology, (i) reprogramming of energy metabolism and (ii) evading immune destruction. In total they describe 10 different mechanisms of tumor biology. The feature of this overarching and integrating concept is its holistic view on a tumor as an organ [1]. Most importantly this concept emphasizes the tumor´s constitution of different cell types with distinct functions in its biology. These specialized cell types build a cellular tumorigenic network and they serve at different steps during the course of tumorigenesis in an integrated inter- and intracellular signaling network. Apart from the neoplastic founder cell which likely originated from some critical event of genetic alteration [2], non-neoplastic cells such as cancer associated fibroblasts, endothelial cells and immune cells comprise the heterotypic cell biology of a tumor. Tumor cell signaling reprograms these cell types leading to induction of processes such as angiogenesis, apoptosis inhibition, immune evasion and synthesis of soluble tumor microenvironment components. During the evolving tumor development signaling networks within the tumor are reshaped further and expression programs such as hypoxia lead to heterogeneity within specific tumor areas which also can be observed by histopathological analysis. Depending on the tumor region and the level of oxygen and nutrients available in designated areas, specific genes are expressed varying from cell type to cell type. In turn, this leads to distinct effects driving the rates of proliferation, vascularization, inflammation and invasiveness. This review will focus on evolving treatment options for non-small cell lung cancer (NSCLC) with respect to the intercellular interdependence within the cellular tumorigenic network. Lung cancer is the leading cause of cancer-related mortality worldwide. Histopathological grading identifies ~80-85% of lung cancers as NSCLCs and 15-20% as small-cell lung cancers (SCLCs) [3]. Most cases of NSCLC are diagnosed at advanced metastasized stages. These tumors and some of non-resectable stage III carcinomas are treated with a platinum based combination chemotherapy which is the mainstay regimen in the absence of predictive driver mutations. First-line chemotherapies for NSCLC therapy consist of platinum-based drugs (carboplatin or cisplatin) combined with cytotoxic drugs such as docetaxel, paclitaxel, gemcitabine, vinorelbine or pemetrexed [4]. In addition, many different targeted therapy drugs are under development or have already been approved for the treatment of NSCLC patients in selected tumor conditions as partly discussed below [5, 6]. However, the 5-year survival rate of NSCLC patients is still below 20% and thus the medical need for the development of effective treatment concepts remains one of the greatest challenges for health care systems worldwide so far [7].

## CURRENT DEVELOPMENTS IN THE TREATMENT OF ADVANCED NSCLC

### Targeted therapy

Upon diagnosis, approximately 50% of all patients with NSCLC present with locally advanced, unresectable or metastasized disease. NSCLC has long been considered as comparatively chemoresistent and the median for overall survival is 10-12 months for platinum based combination regimens with median progression free survival (PFS) of 5 months and remission rates of 15-20% [8, 9]. The establishment of second line regimens introduced a further median PFS of 3-4 months with median overall survival of 7 months following progress on first-line chemotherapy [10, 11]. Since introduction of angiogenesis inhibitors in addition to chemotherapy in the first-line treatment of NSCLC improvements have been made in PFS of unselected patients by approximately 2-3 months, however with conflicting results on overall survival [12, 13]. Two trials that investigated VEGFR-inhibition in addition to docetaxel in second-line treatment of patients with NSCLC led to improvements of median overall survival of 1-2 months versus control [14, 15].

Today it is widely accepted that cancer stem cells (CSCs) carrying oncogenic and tumor suppressor mutations drive the process of tumor progression and cancer risk has been attributed to the number of stem cell divisions [16]. Molecular diagnostics have identified so called “driver mutations” which account in some well-defined populations, for development of lung tumors. K-RAS and epidermal growth factor receptor (EGFR) are the earliest identified driver oncogenes. EGFR is overexpressed in about 50% of NSCLC tumors and correlates with poor prognosis [17]. This observation led to the development of small molecules targeting the intracellular tyrosine kinase domain of EGFR in order to block the abundant downstream signaling in these tumors. Gefitinib and erlotinib were the first drugs with such a mechanism of action that received FDA approval for patients with advanced NSCLC stages IIIB/IV in 2003 and 2004 respectively. However, a response rate of only 10% was observed in early clinical trials with these compounds leading to further investigation of the EGFR signaling pathway in NSCLC [18]. Specific mutations within the EGFR coding gene were observed only in patients responding to EGFR inhibitor therapy. These predictive EGFR mutations occur in about 10-15% of NSCLC adenocarcinoma patients in the Caucasian and in about 30-40% in the East Asian population, preferably in patients who are nonsmokers or former light smokers [19]. The identified predominant EGFR mutations are in-frame deletions in exon 19 (45%), mutations in exon 21, mainly L858R (40-45%) and mutations in exon 18 and 20 [20]. The availability of EGFR-tyrosine kinase inhibitors such as gefitinib, erlotinib and afatinib introduced one further line of therapy for patients with NSCLC and activating mutations in the EGFR gene. The expected median survival of patients with activating mutations is now in the range of about 28 months with median freedom from progression of about 10-12 months [21, 22].

Similar to EGFR, today many different genetic mutations in NSCLC and in other tumor entities have been described and serve as starting point for the development of new compounds targeting hyper-activated kinases. Additional genetic mutations identified in NSCLC adenocarcinoma encompass KRAS (~30%), EML4-ALK (~5%), MET (HGF, ~4%), BRAF/PIK3CA (~3%), HER2/MEK (~2%), ROS1 (~2%) and others with lower incidences. However, it is still remarkable that in NSCLC in about 20% (squamous carcinoma) to 40% (adenocarcinoma) no driver mutations could be identified despite routinely used molecular diagnostics [23, 24]. One of the most frequently encountered oncogenes in NSCLC is K-RAS which has been difficult to address pharmacologically and which is considered one of the cardinal routers of mitogenic signaling. To address K-RAS mutated carcinoma downstream targets have been exploited. For example, MEK was addressed in K-RAS mutated NSCLC tumors by combining the MEK-inhibitor selumetinib with docetaxel in second line therapy. However, this study failed to reach the primary endpoint of progression free survival [25]. In melanoma the addition of a MEK inhibitor, trametinib, to RAF inhibitor dabrafenib improves progression free survival by about 2 months [26].

All therapeutics that were developed for targeting driver mutations in NSCLC, such as gefitinib or afatinib for EGFRm or crizotinib and ceritinib for EML-4-ALK translocation have in common that after a certain time of partial or even complete responses in these patients, resistance mutations evolve and a loss of efficacy for the respective drug is observed. The approximate time until a drug resistant clone is outgrown and mediates therapy resistance in these tumors is less than a year [21, 22].

As a consequence, 2nd and 3rd generation tyrosine kinase inhibitors (TKIs) are being developed targeting the initial driver mutations but circumventing drug resistance mutations. An example of such a drug is osimertinib. Osimertinib is an irreversible 3rd generation TKI approved for treatment of EGFR mutated NSCLC tumors harboring the T790M drug resistance mutation, which evolve in about 60% of EGFRm tumors treated with 1st or 2nd generation TKIs [27]. From this aspect the initial treatment of a tumor harboring a targetable driver mutation reflects the competition against the evolution of drug resistant clones. This results in sequential therapy regimens, trying to stick with the next round of an evolutionary race. However, the development of these drugs has provided a significant step forward in the treatment of many tumor entities. The interim analysis of a sub-cohort of a clinical phase I trial (AURA) with osimertinib in EGFR mutated, but not T790M positive tumors, revealed an objective response rate (ORR) of 67-87% and a median PFS of 19.3 months with a manageable safety profile [28]. Compared to the efficacy of 1st or 2nd generation TKIs in this patient population this is an increase of about 9 months in PFS if the final analysis will confirm these results. The progress made with such drugs for late stage NSCLC therapy becomes even clearer when TKI therapies are compared to platinum-based chemotherapies approved for this indication, resulting in ORRs of about 20% and PFS rates of about 4 months. At the same time the safety profiles of new generation TKIs is more favorable compared to classical chemotherapeutics. For example, in second line NSCLC patients treated with osimertinib the incidence of any adverse event of grade 3 or higher was 32% [29] compared to an incidence of 76% in such patients when treated with docetaxel chemotherapy [10].

### Immunotherapy

Another evolving field in targeted cancer therapy is the therapeutic intervention in tumor biology aiming at the detection and destruction of tumor cells by the immune system. Vaccination with tumor-specific antigens is one approach that is being evaluated since many years. Currently, for NSCLC different vaccines based on tumor antigens including MAGE-3, MUC1 or NY-ESO-1 are in advanced clinical development phases. In addition, cellular based therapy approaches by direct administration of T cells or dendritic cells stimulated with such antigens are being developed. These type of immunotherapies have been shown to prolong PFS and OS in NSCLC patients significantly when compared to treatment arms heterogeneously composed of only placebo, best supportive care or chemotherapy. The cellular approaches were observed to be more effective than vaccination with tumor antigens [OS: HR 0.81, 95% CI, 0.70 to 0.94, *P* = 0.01; PFS: HR 0.83, 95% CI, 0.72 to 0.95, *P* = 0.006] as calculated by a meta-analysis [30].

In this review we will focus on the role of intercellular interactions by immune checkpoint inhibitors as the most impactful development in immunotherapy within the last decades. The underlying concept of this approach is the cancer immune-editing process. Three distinct phases of this process of intercellular signaling lead to the evasion from immune detection and the final outgrowth of the tumor [31]. In the first phase, the elimination phase, transformed cells are still well detected by the collaboration of the adaptive and the innate immune system resulting in their elimination. This phase is driven by immune stimulatory signals of innate danger signals, tumor antigens and NKG2D ligands. Possibly this phase may be survived by transformed cells that are less immunogenic and cells that have the ability to modulate their cellular microenvironment by intercellular signaling impairing the adaptive immune system. T-cells, INF-γ and IL-12 have been implicated to influence the functional dormancy of transformed cells in this phase [32]. Continuous evolutionary pressure on transformed and genetically instable cells in the equilibrium phase is likely to result into the escape phase. Therein specific clones of transformed cells which failed to be recognized by the adaptive and innate immune system dominate the cellular network. Signals from this network maintain an immunosuppressive cellular tumorigenic network and enable the outgrowth of this cellular complex as a clinical significant tumor [33]. During the last decade some of the main drivers of this immune escape process have been identified and became well known as so called immune-checkpoint inhibitors. Among them cytotoxic T-lymphocyte-associated antigen 4 (CTLA-4) was the first molecule found to be expressed on T-cell subsets as a downmodulator of their activation. This intercellular signaling firstly requires T-cell receptor (TCR) activation on a T-cell by antigen uptake and interaction of the TCR with a MHC-I molecule on an antigen presenting cell (APC) bringing both cells into spatial proximity. At the same time a CD28 molecule on the T-cell engages with a CD80 (B7.1) and a CD86 (B7.2) molecule further increasing the activation of the T-cell by the initial TCR-MHC-I interaction. Subsequently CTLA-4 is becoming expressed on the surface of the T-cell and competes for binding to CD80 and CD86 with CD28 resulting in a counter signal that prevents an over activation of the immune cell. CTLA-4 has been clinically demonstrated to be involved in the process of the immune-editing escape phase by mediating immune suppression on T-cell activity and thus preventing an effective adaptive immune response against tumor cells in different tumor entities [34]. In line with these results a recombinant therapeutic protein encompassing the extracellular domain of CTLA-4, abatacept, has been FDA-approved for the immunosuppressive treatment of rheumatoid arthritis [35]. Ipilimumab was the first therapeutic antibody against CTLA-4 approved for treatment of malignant melanoma. In a clinical study ipilimumab showed a long-term survival for more than two years in 18% of patients in a study population without any further treatment options except experimental therapy. This result showed for the first time an exceptional long duration of response after short treatment periods when compared to non-immune based therapies in oncology. This finding underscores an induced and direct effect of the adaptive immune system as a mechanism of this therapy concept. However, immune-related toxicities frequently occurred in ipilimumab treated patients and required an exceptional level of attention [36].

A second and more recently discovered mechanism of immune-checkpoint inhibition is the interaction of the molecules PD-1 on T-cells (predominantly CD8+) and PD-L1 on APCs such as tumor cells, dendritic cells, cancer associated fibroblasts and immune cells expressed in 20%-50% of human tumors [37]. The PD-1/PD-L1 interaction prerequisites the same process of TCR-MHC-I interaction as described above for CTLA-4 and also functions as an inhibitory signaling for T-cell activity when established. In contrast to the predominant localization of CTLA-4 in lymphatic tissue, the immunosuppressive role of PD-1/PD-L1 engagement seems to be critical directly within or in close proximity to the cellular tumorigenic network forming the tumor [38].

So far, encouraging clinical activity of anti-PD-1 antibodies was observed in many different tumor entities including lung, colon, head and neck, gastric, melanoma and renal cell carcinoma [39, 40]. The first indication a PD-1 checkpoint inhibitor received FDA approval was metastatic melanoma where nivolumab showed superiority versus chemotherapy with an ORR of 40% versus 13.9% and an overall survival rate (OS) of 72.9% versus 42.1% [41]. For the anti-PD-1 antibody pembrolizumab an ORR of up to 38% was found in metastatic melanoma patients [42]. In the second line treatment for advanced NSCLC of squamous histology (Checkmate 017 trial, Phase III) or of non-squamous histology (Checkmate 057 trial, Phase III) ORRs of 20% versus 9% (squamous) and 19% versus 12% (non-squamous) were observed for nivolumab when compared to docetaxel. When comparing nivolumab with docetaxel median overall survival times were significantly improved from 9.2 versus 6.0 months [squamous histology; HR 0.59; 95% CI, 0.44-0.79; *P* < 0.001] and 12.2 versus 9.4 month [non-squamous histology; HR 0.73; 95% CI, 0.59-0.89; *P* = 0.002]. Remarkably the median duration of response in the non-squamous study population was 17.2 months compared to 5.6 months for nivolumab and docetaxel respectively. In general, in both studies fewer side effects were observed for patients treated with nivolumab [43, 44]. Patients in these trials were not stratified for PD-L1 expression. These results led to recent FDA approval of nivolumab as second line therapy for the treatment of non-squamous and squamous advanced NSCLC without mandatory PD-L1 expression analysis.

Another phase II/III randomized trial assessed second line treatment with pembrolizumab versus docetaxel in a population with advanced squamous or non-squamous NSCLC stratified for PD-L1 expression ≥1%. Median OS was significantly longer in the pembrolizumab study population with 10.4 months [HR 0.71; 95% CI, 0.58-0.88; *P* = 0.0008] and 12.7 months [HR 0.61; CI, 0.49-0.75; *P* < 0.0001] for 2 mg/kg and at 10 mg/kg pembrolizumab, respectively with 8.5 months for docetaxel. Tumors with PD-L1 expression ≥50% showed a significantly increased overall survival in the populations treated with pembrolizumab compared to docetaxel [2 mg/kg: 14.9 vs 8.2 months; HR 0.54; 95% CI, 0.38-0.77; *P* = 0.0002 and 10 mg/kg: 17.3 vs 8.2] [45].

The most impactful development in the field of immune checkpoint inhibitors is the approval of pembrolizumab for first line therapy in PD-L1 positive NSCLC patients based on results from the keynote 024 study. This study found a significantly prolonged progression free survival of 10.3 months in pembrolizumab treated patients versus 6 months in patients treated with a platinum-based chemotherapy [HR for disease progression or death, 0.50; 95% CI, 0.37-0.68; *P* < 0.001]. Also in terms of response to treatment pembrolizumab showed superiority versus chemotherapy with an ORR of 44.8% versus 27.8%. OS was significantly longer in pembrolizumab than in chemotherapy treated patients [HR for death 0.60; 95% CI, 0.41-0.89; *P* = 0.005]. Due to these impressive results the study was stopped preliminary and patients in the chemotherapy arm were offered treatment with pembrolizumab [46].

The first FDA approval of a PD-L1 inhibitor for NSCLC patients who progressed on a platinum-based chemotherapy was granted for atezolizumab recently [47]. This approval is based on results from the POPLAR phase II and the OAK phase III studies. The median OS in POPLAR was 12.6 months in the atezolizumab arm compared to 9.7 months in the docetaxel arm [HR 0.73; 95% CI, 0.53-0.99; *P* = 0.04] [48]. The OAK study showed a median OS of 13.8 months in the atezolizumab arm versus to 9.6 months in the docetaxel arm [HR 0.74; 95% CI, 0.63-0.87; *P* = 0.0004] [49].

Taken together, to date both anti-PD-1 antibodies, pembrolizumab and nivolumab and the PD-L1 antibody atezolizumab are FDA-approved for the treatment of advanced squamous and non-squamous NSCLC for second line therapy. Currently pembrolizumab is the only checkpoint inhibitor approved for first line treatment of NSCLC patients. For treatment with pembrolizumab the assessment of PD-L1 expression in ≥50% of tumor tissue by a companion diagnostic biomarker test (PD-L1 IHC 22C3 pharmDx kit) is mandatory.

An ongoing effort in the field of immune-checkpoint inhibition is the evaluation of combination therapies of anti-PD-1 or anti-PD-L1 antibodies with anti-CTLA-4 antibodies or chemotherapies [50]. The CTLA-4 antigen becomes recruited to the surface of T-cells upon CD28 activation and limits CD80/86 induced T-cell activation by scavenging CD80/86 from binding to CD28. The rationale for the combination of CTLA-4 and PD-1/PD-L1 inhibition is based on the observation, that CTLA-4 inhibition might be a prerequisite for the release of activated, tumor-antigen specific T-cells from lymphatic tissues capable for the subsequent infiltration of tumor tissue. When these activated T-cells become localized to the tumor, the inhibition of the PD-1-PD-L1 axis upon activated TCR-MHC-I interaction between T-cell and tumor cells enables an efficient, localized immune response against tumor cells [38]. Ipilimumab is currently being trialled in combination with anti-PD-1 antibodies in first line treatment of lung cancer and preliminary phase I data suggest a clinical efficacy no less than a PD-1 single agent or chemotherapy combination approach in this setting with unprecedented survival rates [51–55]. The potential synergies of immune checkpoint inhibition and activation of T-cell responses by vaccination or by cellular therapies presenting tumor specific antigens at the same time are currently under investigation in clinical trials [56].

## INTERDEPENDENT CROSSTALK OF THE CELLULAR TUMORIGENIC NETWORK

As described by Hanahan and Weinberg in 2011 and discussed above, a cellular tumorigenic network consists of different classes of cells [1]. Tumor endothelial cells (TECs), tumor cells (TCs) and immune cells (ICs) interact with the stromal compartment during the evolution of the tumor in an integrated inter- and intracellular signaling network [57]. An increasing body of evidence is provided that this interdependence could be the initial cause of *de novo* or adaptive drug resistances in many cases [1, 57, 58]. Cell types unaffected by drug treatment may substitute for impaired signal transduction by targeted therapy drugs (see Table 1 for overview).

**Table 1:** Evidence for paracrine resistance mechanisms to targeted therapies of the cellular tumorigenic network in NSCL

Mechanism of action	Observed effect	Evidences for de novo and adaptive resistance mechanisms	Signaling to be addressed for preventing cellular resistances
anti-VEGF/VEGFR	Regression of existing tumor vasculature [61,124] Inhibition of new and recurrent tumor vessel growth [122] Interruption of angiogenic signaling leading to the destruction of tumor vasculature [61, 62]	Increase of hypoxia-induced factors mediating tumor progression and treatment resistance [62,63,93] Hypoxia initiates recruitment of suppressive and proangiogenic ICs and results in upregulation of PD-L1 on TCs and other cell types [66,115] CAFs and TCs substitute for interrupted TEC signaling by secretion of HGF, EGF and SDF-1, by inducing expression of the corresponding receptors and by direct interaction [63,92,97,123] COX2 produced PGE2 is a mediator of resistance to VEGFR-inhibiton [106]	CXCR4/SDF1, bFGF/FGFR, IGF-II/IGFR, HGF/HGFR, COX2/PGE2/EP [58,63,92,105] PD-1/PD-L1 [115] COX2/PGE2/EP [58,95,106]
anti-EGF/EGFR	Inhibition of TC proliferation and induction of TC apoptosis [125]	CAFs and TCs substitute for interrupted EGFR signaling by secretion of HGF and SDF-1, by inducing expression of the corresponding receptors and by direct cell to cell interaction [97,99,100] Expression of CXCR4 in EGFR TKI resistant TCs maintains stemnes and therefore SDF-1 secreting cells such as CAFs and/or TECs may substitute for interrupted EGFR signaling by paracrine signaling via CXCR4 [85] Outgrowth of TC drug resistant subclones (e.g. T790M) and HGFR amplification [27,126]	CXCR4/SDF1, bFGF/FGFR, IGF-II/IGFR, HGF/HGFR, COX2/PGE2/EP [58,95]
anti- PD-1 /PD-L1	T-cell mediated adaptive immune response resulting in apoptosis of TCs [38]	Absence of PD-L1 expression [113] T-cell anergy and CD8+-T cell induced immunosuppression [114] Upregulation of alternate immune checkpoint inhibitors such as TIM-3 and LAG-3 in ICs [127,128]	TIM-3, LAG-3 [128]

Legend TC: tumor cells, CAF: cancer associated fibroblasts, TEC tumor endothelial cells, IC: immune cells, CSC: cancer stem cell

### Tumor endothelial cells (TECs)

The process of angiogenesis is one of the key characteristics of neoplastic growth. The angiogenic switch driven by the onset of hypoxia in newly forming cellular tumorigenic networks locally reinstates the embryogenetic growth program for blood vessels and leads to tumor neovascularization originating from preexisting blood vessels [59]. The initial concept of targeting angiogenic factors for tumor therapy, such as VEGFR2 or its ligand(s) VEGF-(A) was based on the presumption of their almost exclusive expression in tumor tissue. Today several drugs are approved and target VEGFRs (e.g. sunitinib, sorafenib, ramucirumab) or VEGF (bevacizumab). Noteworthy, anti-angiogenic therapy is directed towards endothelial cells but not to tumor cells directly. The drug targets VEGFR-2 and VEGFR-3 are localized primarily to the vasculature in human primary solid cancers but not to the tumor cells [60]. Thus the therapeutic effect of these drugs is anticipated to be based on the interruption of angiogenic signaling in treated tumors, which in turn firstly leads to the destruction of tumor vasculature [61, 62]. The effect on tumor cells seems to be a secondary event based on an increase of hypoxia and nutrient deficiency. It was shown that it is countered by upregulation of growth factors which have the capacity to replace VEGF and stimulate new blood vessel growth such as EGF and SDF1α as well as their receptors [63]. At the same time the destruction of tumor vasculature leads to increased hypoxia in affected areas of the tumorigenic cellular network [64]. In consequence HIF-1α driven survival factors are expressed in hypoxic areas from tumor cells, cancer associated fibroblasts and immune cells and thereby may protect certain subpopulations of tumor cells from apoptosis or necrosis [58, 65, 66].

Mathematical modelling suggests that hypoxia may induce a glycolytic phenotype which is more prone to invasiveness [67]. Taken together the use of angiogenesis inhibitors, based on their primary targeting of tumor endothelial cells but not tumor cells, CAFs or immune cells may initiate a pattern of therapy resistance that either develops after a certain time of therapy (adaptive resistance) or is immediately effective based on the specific tumor type (*de novo* resistance).

### Tumor cells (TCs)

Analysis of tumor cells as the classical target of drug treatment in oncology have revealed a surprising complexity of cancer genomes. These cells harbor driver mutations representing underlying events for tumor initiation and progression. Such as *EGFR KRAS, PTEN* mutations, *PIK3CA* amplifications and *EML4-ALK* translocations in NSCLC [23]. Analysis of both squamous and nonsquamous carcinoma genomes in the TCGA project revealed mutations or amplifications in oxidative stress response pathway NFE/KEAP1, in squamous differentiation related pathway SOX2/TP63, alterations in HLA-A, the multitude of cell-cycle control and p53 pathways as well as nucleosome modelling and RNA processing pathways [68, 69]. Of note, it was stated that genome data did not explain all pathway activation patterns, and many tumors lack genomic alterations to explain phosphoprotein activation [69], a fact that may reflect epigenetic alterations [70]. Accordingly, in 20% of squamous carcinoma and 40% of adenocarcinoma no accountable mutations have been identified so far [24].

Research on signal transduction network dynamics showed that addressing oncogenic targets may result in paradoxical effects and that oncogenicity may be context-dependent [71]. The MAPK/ERK pathway displays a feedback inhibitory loop to upstream located targets such as EGF. Inhibition of MEK relieves this feedback inhibition and renders AKT signaling more active which could explain why simple MEK inhibition did not achieve pronounce changes in progression free survival [71, 72].

The multitude of observed alterations raises the question as to whether such tumor populations could more effectively be addressed by exploiting combinations of agents directed against established pathways attempting to evoke what has been called synthetic lethality and well worked out in the context of ovarian cancer [73]. In NSCLC, the EGF receptor is probably the best investigated and evaluated druggable molecular target and scrutinizing EGFR signaling could help to uncover possible combination regimens. EGFR overexpression is observed in the majority of NSCLC tumors [17]. NSCLC tumor cells interact with their surrounding cellular tumorigenic network via EGFR signaling and other cell surface receptors that contribute to survival, proliferation, induction of tumor promoting factors and immune evasion [74]. The anti-EGFR antibody cetuximab, approved for treatment of EGFR expressing colorectal cancers, competitively blocks the binding of EGF to its receptor. As a consequence, paracrine signaling within the cellular tumorigenic network by EGF originating from CAFs and immune cells for example, is blocked by EGFR signaling interruption. In addition, cetuximab is capable of depleting tumor cells by antibody depending cytotoxicity [75].

In NSCLC adenocarcinoma with activating EGFR mutations, such as L858R and Del19 the receptor seems to be constitutively active and independent from ligand binding [76] resulting in what has been termed oncogene-addiction [77]. Signal withdrawal evokes large shifts in the apoptotic balance resulting in the rapid and extensive remissions typically observed following EGFR-TKIs in these cancers. In squamous lung cancer, however, almost no such activating mutations have been observed albeit a rate of 7% overexpression of EGFR [68]. A recent phase III trial in patients with squamous cell carcinoma of the lung and selected for EGFR overexpression but not for EGFR mutation revealed a significantly improved overall survival of 11.8 months in patients receiving the EGFR-mAb necitumumab plus gemcitabine-cisplatin versus 10 months in the gemcitabine-cisplatin control group [HR 0.79; 95% CI, 0.69- 0.92] [78]. This observation provides evidence, that also in non-EGFR mutated tumors targeting of EGFR signaling has an impact on the clinical outcome.

Activating EGFR mutations display a shift towards stronger STAT3 and STAT5 signaling [79, 80]. This suggests that STAT3 induced genes that shift the immune balance towards a suppressive, tolerogenic environment could be addressed to revert this effect. The precise timing of STAT3 modulation or inhibiton has not been worked out and it is known that persistent STAT3 ablation may cause autoimmunity in mice [81]. This points to the possibility of intermittent or chronometric dosing of agents that interfere with STAT3 signaling. For activation of STAT3 both EGFR mediated signaling as well as IL-6 signaling has been shown to be relevant and in NSCLC tumor cells JAK1 and not JAK2 was determined as the signal relaying kinase [82]. Ruxolitinib and Tofacitinib, JAK-inhibitors that can cover all three JAK-kinases, are possibly suited to suppress STAT3 mediated effects in a two pronged way that includes EGFR blockade. Still, it is unclear whether JAK-inhibition will result in a clear improvement of the immunosuppressive environment. IL-6 is one of the major effectors of immunosuppression and a target of STAT3 [81]. Therefore, IL-6 inhibitors, either chronometrically or continuously dosed could be an option to further improve freedom from progression in these patients. Currently approved antibodies against the IL-6 receptor or IL-6 itself are tocilizumab and siltuximab, respectively. Both are being trialled in solid tumor indications and a role of siltuximab in suppressing IL-6 mediated STAT3 activation has been described [82].

Receptor tyrosine kinase signaling including EGFR is associated with the emergence of epidermal-mesenchymal transition (EMT) which is considered a central switch to invasion and metastasis [71]. EMT is a complex process the current understanding of which does not yet allow a meaningful approach and its dynamics suggests a gradual shift with some tumor cells still with epithelial characteristics and some with transitional or mesenchymal phenotype which may imply an altered driver kinase dependency in the context of the cellular tumorigenic network.

The main cause of treatment resistance is the emergence of resistant tumor cells after therapy, described in many different tumor entities [83]. Cancer stem cells (CSCs) are suspected being the origin of outgrowing therapy resistant tumor cells. CSC have a high capacity for self-renewal and multilineage differentiation and are believed to be responsible for tumorigenesis, therapeutic resistance, metastasis and recurrence of cancer [84]. In NSCLC tumor cells resistant to the EGFR TKI gefitinib the expression of CXCR4 maintains stemness through JAK/STAT3 downstream signaling. In these drug resistant tumor cells the CXCR4 inhibitor plerixafor (AMD3100) exhibits significant anti-tumorigenic effects [85]. Most interestingly Phillip *et al*., showed that activation of EGFR by its ligand under hypoxic conditions enhances CXCR4 expression leading to malignant transformation through increased proliferation, survival and motility [86]. Treatment with plerixafor is also capable to sensitize prostate cancer cells and pancreatic cancer cells to chemotherapy [87, 88] and it is believed that attachment of CXCR4 expressing cells to the ECM protects cancer cells from chemotherapy. In line with these results anti-tumor activity also was observed for the SDF-1 peptide analogue CTCE-9908 in different tumor entities including breast and prostate cancer [89–91]. CTCE-9908 was shown to enhance the efficacy of anti-VEGF treatment in an experimental mouse model [90] consisting with the observation that a high expression of CXCR4 was found to correlate with insensitivity against treatment with the VEGR inhibitor sunitinib [92]. Tumor cells are also tightly linked to tumor endothelial cells since they are the main source of VEGF secretion inducing and maintaining angiogenesis by paracrine signaling [64]. Anti-angiogenesis therapy, such as bevacizumab treatment, has an indirect effect on tumor cells as described above but leads to the activation of the hypoxia HIF-1α program resulting in the upregulation of drug resistance pathways [62, 63, 93].

### Cancer associated fibroblasts (CAFs)

The connective tissue of the tumor microenvironement consists of the extracellular matrix (ECM), and fibroblasts of which cancer-associated fibroblasts (CAFs) as a distinct class are being discerned from tissue-associated native fibroblasts [57]. CAFs are thought to derive from epithelial and endothelial mesenchymal transitions, from myeloid precursors and from host fibroblasts [57, 94]. They are thought to coevolve with the tumor and migrate, differentiate and secrete factors which may influence both tumor cells as well as the surrounding immune cells [94]. Their role and their contribution to tumorigenesis, angiogenesis and invasiveness within the cellular tumorigenic network became increasingly recognized during the last years [58]. Increasing evidence is provided that CAFs provide a target for chemoprevention in lung cancer and other tumor entities [95, 96].

CAFs orchestrate the cellular tumorigenic network and contribute to cancer stemness by secreting growth and survival factors such as SDF-1, bFGF, IGF-II and HGF and by inducing the expression of their respective receptors CXCR4, IGF-1R, HGFR [85, 97, 98]. Evidence is provided that CAF secreted HGF provides resistance to EGFR TKI therapy in NSCLC patients by cytokine crosstalk [99] and CAF subsets mediate *de novo* resistance to EGFR TKI therapy in *EGFRm* NSCLC [100]. Several of these factors are also known to be HIF-1 regulated and become increasingly expressed in different cell types in hypoxia. CAFs were also shown to modulate the tumor microenvironment by the expression of VEGF-A [101] and EGF [58] and thus contribute to angiogenesis. Among the CAF secreted cytokines the SDF-1/CXCR4 axis is best investigated. SDF-1 of fibroblast origin can promote tumor growth and angiogenesis [102]. CXCR4 expression on tumor cells has been associated with a negative prognostic value for survival, homing to tissues with SDF-1 expression, resistance to angiogenesis inhibition and resistance to chemotherapy [91, 103].

High expression of CXCR4 also correlates with insensitivity against treatment with the VEGR inhibitor sunitinib in renal cancer [92]. In addition, CAFs also display a proinflammatory gene signature mediated by NF-kB signaling [104]. Together with the cancer cell they are a source for immunosuppressive factors like IL-6, IL-11, COX2 and also for VEGF, all of which are STAT3 induced as well as being inducers of immunosuppressive STAT3 signaling [81]. Expression of COX2 and PGE2 within the cellular tumorigenic network has been shown to increase tumor invasiveness and function as mediators of tumor progression [105]. COX2 produced PGE2 was recently shown to be a mediator of resistance to the VEGFR-inhibitor axitinib and the combination of axitinib and COX2 inhibition was suggested to be a potential target to suppress metastasizing potential [106, 107]. IL-6 secreted by CAFs mediates EMT and contributes to platinum resistance in NSCLC cell lines and isolated cancer cells of NSCLC patients [108].

### Immune cells (ICs)

Immune cells have long been known to play an important role in tumorigenic processes. Inflammatory cells infiltrating the tumor contribute to angiogenesis, tumor cell proliferation and metastasis. These effects are based on signaling within the cellular tumorigenic network by the release of factors such as VEGF, EGF, cytokines and chemokines [57, 100, 110]. Despite the potential localization of inflammatory cells and their pro-inflammatory effects within this network, such as NK cells and CD8+ CTLs, neo-antigens from tumor cells are rarely recognized due a state of immunosuppression. Tumor cells express factors that inhibit an anti-tumor immune response, such as programmed cell death 1 ligand 1 (PD-L1), indoleamine 2,3-dioxygenase (IDO), IL-10 and transforming growth factor-β (TGF-β). The immunomodulatory effects of these factors prevent the process of antigen recognition, CTL activation and immune response towards antigen-expressing cells [111]. Tumor-specific antigens may originate from oncogenic viruses, differentiation antigens and epigenetically modified molecules or from mutation-induced neo-antigens [112]. As described above the underlying mechanism of the suppression of T-cell effector functions is based on constitutively overexpressed immunosuppressive cell surface molecules on tumor cells, such as PD-L1 [38]. Breaking the immune-checkpoint inhibition within the cellular tumorigenic network is currently one of the most promising therapy concepts in oncology. However, patient populations with NSCLC tumors driven by EGFR mutations or EML4-ALK translocations treated with PD-1/PD-L1 inhibitors showed reduced ORRs compared to patient populations harboring EGFR and ALK wild-type tumors. This observation is in line with low rates of PD-L1 expression and CD8+ tumor-infiltrating cells (TILs) in corresponding tumors [113]. In T-cell infiltrated tumors with a signature indicative of active Th1-type response tumor escape is characterized by PD-L1 upregulation, by induction of and infiltration with CD25+FOXP3+Tregs and by T-cell anergy characterized by defective IL-2 secretion upon antigen stimulation [114]. An antibody against CTLA-4 in combination with anti PD-L1 was shown to revert T-cell anergy in this setting as shown by increased proliferation and increased production of IL-2 and TNF-α indicative of functional T cells [114]. Most interestingly PD-L1 has recently been described as a direct target of HIF-1α consisting of an active HIF response element (HRE) in its promotor region. Experiments in tumor-bearing mice under hypoxic conditions resulted in a significant up-regulation of PD-L1 on macrophages, dendritic cells, and tumor cells [115]. This observation underscores the multiplicity of pathway interactions and their interdependencies between the components of the cellular tumorigenic network once again. Still, remission rates induced by PD-1 antibody nivolumab second line after platinum based chemotherapy in both squamous and non-squamous carcinoma of the lung are in the range of 20% with a median PFS in the range of 2.2-3.5 months [43, 44].

In the cellular tumorigenic network, the balance between tumor cells and the cells of the surrounding innate and adaptive immune system is determined by elements with both tumor repressive and tumor supportive features. The cell types that act in a tumor-repressive immunogenic way are governed by NK/NKT-cells and Th1-cells. Macrophages and Neutrophils display type 1 characteristics and IFNγ, IL-2 and IL-12 are important cytokines [116]. The tumor promoting arm of that balance is determined by the presence of cells of myeloid origin, Type 2 suppressive T-cells (Tregs) and Th2-cells and IL-4, IL-6, IL-10 and IL-13 are relevant cytokines. The ratio of Th2/Th1 cells correlates with parameters of clinical progression in breast cancers [116]. The role of Th17-cells is considered to be context dependent and can be both repressive and supportive [57, 109, 110, 116]. STAT signaling plays a major role in the crosstalk between somatic and immune cells and STATS 1, 2 and 4 contribute to a tumor suppressive response whereas STATs 3, 5 and 6 act more immunosuppressive [81]. Tumor cells are able to induce a suppressive phenotype by STAT3-signaling which leads to secretion of IL-6 and may suppress the activity of several cell types relevant to antigen presentation and cytotoxic T-cell responses. Numerous genes are being influenced by STAT3. They include proliferation and survival related genes and angiogenesis genes like VEGF, HIF1α, bFGF and HGF as well as immunosuppressive factors like IL-6 and IL-10 [117]. STAT3 also suppresses the expression of Th1-associated gene products like IFNb, IFNγ and IL-12 and induces genes like COX2 and NOS that are associated with an inflammatory phenotype. IL-6 induced genes may themselves induce STAT3 and thus contribute to maintenance via a feedforward loop [118]. COX2 as a STAT3 induced gene is by itself a suppressor of antigen-specific immunity. Local overexpression of COX2 generates an immunosuppressive milieu by mechanisms that involve IL-4, IDO and IFNγ [119]. PGE2 as a product of COX2 amplifies the suppressive activity of CD25+ Tregs and induces FOXP3+ in both CD25- and CD25+-cells thus contributing to immunosuppression [120]. In NSCLC a large share of tumor infiltrating leukocytes are CD25+ Tregs that may suppress T-cell proliferation. An increased number of CD4+FOXP3+ Tregs correlates with poor prognosis in NSCLC suggesting that differentiation of CD4+ to CD4+FOXP3+ cells may contribute to tumor escape. Thus, influencing factors such as COX2 and IL-6 display effects on tumor progression.

## THERAPEUTIC APPROACHES FOR DISRUPTING KEY SIGNALING CROSSTALK IN THE CELLULAR TUMORIGENIC NETWORK OF NSCLC

As described above the cellular tumorigenic network can be described as an interdependent network of intercellular signaling via cell surface receptors of its different cell types. As an example in case of *EGFRm* NSCLC these dependencies are profoundly skewed in favor of the EGFR as a driver stimulus. Recently the combination therapy of bevacizumab plus erlotinib was approved for first line therapy of patients with EGFR mutated advanced NSCLC based on a Japanese clinical phase 2 study. In this trial, median progression free survival in the combination arm was 16.0 months (95%CI 13·9-18·1) versus 9.7 months with erlotinib alone [HR 0.54; 95%CI, 0·36- 0·79]; log-rank *P* = 0·0015). Three complete responses (CRs) were observed in the combination arm versus one CR in the erlotinib monotherapy arm. Interestingly, overall response rates of 69% in the combination arm compared to 64% in the monotherapy arm were not significantly different. The incidence of grade 3-4 adverse events was elevated in the combination arm. However, no new AE categories were identified and the safety profile was manageable in both groups [121]. For the first time these results show a significant additive effect in the treatment of advanced NSCLC patients by combining the two different treatment concepts of anti-angiogenesis and inhibition of tumor cell proliferation based on *EGFR* driver mutations. Because the combination therapy only had a minimal effect on the initial response rates (ORRs 69% versus 64%), blocking of both mechanisms must have had at least an effect on the adaptive cellular resistance preventing the outgrowth of erlotinib drug resistant tumor cell subclones for a longer time in the combination arm than under erlotinib monotherapy. Obviously, in this setting of a kinase-addicted tumor the addition of bevacizumab to erlotinib potentiated the effect of erlotinib, resulting in what can be called an evolutionary bottleneck to the tumor in its cellular tumorigenic network. For this combination, one may speculate that after an initial depletion of tumor cells by erlotinib outgrowth of drug resistant tumor cell clones, harboring resistance mutations such as T790M was prevented by simultaneous inhibition of tumor endothelial cell growth and their secretion of pro-tumorigenic factors, such as SDF-1, by bevacizumab [65, 122]. This effect may have prolonged time to an adaptive cellular resistance. A later occurring loss of efficacy in the combination arm may have been based on a slowly evolving substitution of tumor endothelial cell signaling by CAFs or immune cells within the cellular network. Evidence for a similar mechanism has recently been provided showing that a combined suppression of endothelial cells and CAF growth resulted in synergistic effects when using bevacizumab in bevacizumab-resistant cancer cells (Table 1) [123].

**Figure 1:**
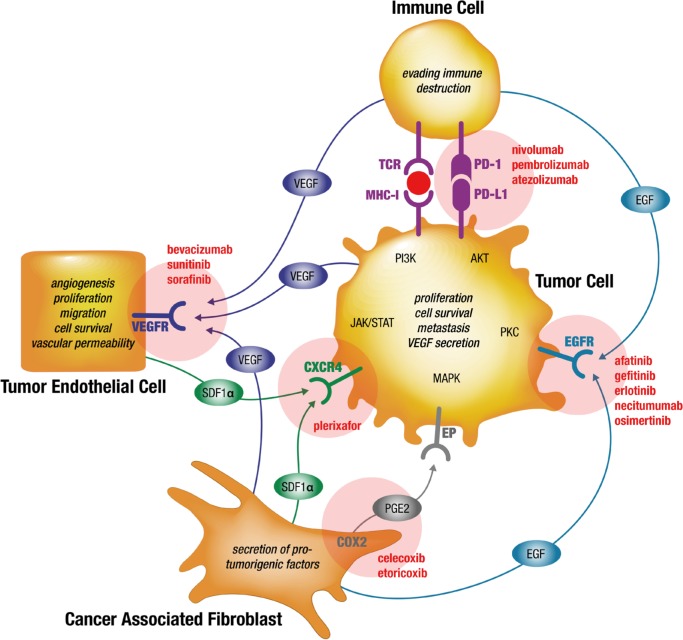
Simplified model of preventing drug resistances by simultaneous targeting of interdependent signaling in the cellular tumorigenic network of NSCLC tumors using already marketed drugs Signaling axes of VEGF-VEGFR, EGF-EGFR, SDF-1-CXCR4, COX2-PGE2-EP and PD-1-PD-L1 are exemplary shown for some of the known paracrine pathways binding the cellular tumorigenic network in NSCLC tumors. Evidence is provided that resistances to targeted therapy drugs is partly based on substitutions of inhibited pathways in monotherapy. Therefore, combined targeted therapies against selected pathways may overcome primary and secondary drug resistance. Examples of FDA-approved drugs are provided adjacent to the respective targeted pathways (updated from Langhammer, 2013 [129]). PGE2, prostaglandin E2; VEGFR, vascular endothelial growth factor receptor; EGFR, epidermal growth factor receptor; COX2, cyclooxygenase 2; red circle: tumor neo-antigen.

Taken together the study published by Seto *et al*., confirmed the pronounced efficacy of EGFR TKIs in NSCLC patients harboring EGFR mutations and for the first time showed that a meaningful improvement of this therapy concept is possible by combining drugs with different mechanisms of action. This effect might be expected of similarly kinase-addicted types of tumors such as those with activating translocations of ALK and ROS. This observation provides the rationale for further combinations of targeted therapies addressing the factors of interdependency in the cellular tumorigenic network as discussed in this review and which may lead to improved therapeutic efficacies. Therefore, a combination therapy should address each cell type involved in linking the cellular tumorigenic network: The proliferation of malignant tumor cells can be effectively targeted at their driver mutations if present, such as for EGFR mutated tumors by EGFR TKIs. The tumor promoting effects of tumor endothelial cells (angiogenesis, tumor cell survival and metastasis) can be targeted by inhibition of VEGFRs or VEGFs. Mediated survival signals to tumor cells originating from cancer associated fibroblasts can be targeted by blocking the SDF-1-CXCR4 axis and COX2 for example. At the same time immune cell activation could further elevate the pressure on the cellular tumorigenic network integrity by PD-1 or PD-L1 inhibition. Against all of these different tumor targets approved drugs are available (Table 1; Figure 1). Concerns regarding the potential toxicity of combination therapies should be taken into account. Start dosages of selected combined drug regimens may be chosen in dose escalation steps starting significantly below monotherapy dosages, anticipating additive or synergistic effects and thus lowering toxicity at the same time. Patient stratification should be mandatory by expression (e.g. PD-L1, CXCR4, COX2) or mutational (*EGFRm*) analysis in tumor biopsies.

In the interest of a valid clinical research rationale, it would be most desirable that selected targets for the design of novel therapeutic approaches possess predictive values. In this approach it holds true for PD-L1 expression and for EGFR mutations as described above, but it remains less clear for CXCR4 and COX2. However, in line with the presented data-based hypothesis the predictivity of some targets may only be observable in combinational therapeutic approaches.

In summary, the results reviewed in this article provide evidence that simultaneous and distinct targeting of signaling from different cell types forming the cellular tumorigenic network may break the intercellular crosstalk and thus may overcome *de novo* and may delay adaptive drug resistances. Noteworthy, the direct suppression of cellular interdependency may most likely be achieved when targeting paracrine signaling axes of cell surface receptors and their respective ligands critical for the respective tumor biology. Based on the findings discussed in this review we suggest the following five criteria for selecting a combinational targeted therapy approach in late stage NSCLC patients:

- Target expression or activating mutation proven by expression and/or mutational analysis
- Target should be part of a paracrine signaling pathway mediating intercellular interdependency within the cellular tumorigenic network (see Table 1)
- Drug combination should be selected from non-overlapping intercellular signaling axes targeting each cell type of the cellular tumorigenic network
- Selected drugs should have proven anti-tumor activity in clinical or preclinical studies
- Manageable safety profile of drug combinations.
